# The Acute and Residual Effect of a Single Exercise Session on Meal Glucose Tolerance in Sedentary Young Adults

**DOI:** 10.1155/2012/278678

**Published:** 2012-05-16

**Authors:** Kevin R. Short, Lauren V. Pratt, April M. Teague

**Affiliations:** Section of Diabetes and Endocrinology, Department of Pediatrics, University of Oklahoma Health Sciences Center, 1200 Children's Ave, Suite 4500, Oklahoma City, OK 73104, USA

## Abstract

The study goals were to (1) establish the variability in postprandial glucose control in healthy young people consuming a mixed meal and, then (2) determine the acute and residual impact of a single exercise bout on postprandial glucose control. In study 1, 18 people completed two similar mixed meal trials and an intravenous glucose tolerance test (IVGTT). There were strong test-retest correlations for the post-meal area under the curve (AUC) for glucose, insulin, and Cpeptide (*r* = 0.73–0.83) and the Matsuda insulin sensitivity index (ISI, *r* = 0.76), and between meal and IVGTT-derived ISI (*r* = 0.83). In study 2, 11 untrained young adults completed 3 trials. One trial (No Ex) was completed after refraining from vigorous activity for ≥3 days. On the other 2 trials, a 45-min aerobic exercise bout was performed either 17-hours (Prior Day Ex) or 1-hour (Same Day Ex) before consuming the test meal. Compared to No Ex and Prior Day Ex, which did not differ from one another, there were lower AUCs on the Same Day Ex trial for glucose (6%), insulin (20%) and C-peptide (14%). Thus, a single moderate intensity exercise session can acutely improve glycemic control but the effect is modest and short-lived.

## 1. Introduction

The recent increase in obesity and metabolic disorders in young people highlights the need for more effective lifestyle programs to address current and future disease risk [1–4]. It is well established that physical activity plays a major role in health and metabolic function since sedentary lifestyle and low fitness are risk factors for cardiometabolic disease [5–7]. Further, it is clear that insulin resistance can be reduced by regular exercise even in obese or insulin-resistant people when performed over several weeks or months [8–12]. In adults, there is also evidence that the effect of a single exercise session on insulin sensitivity and glucose tolerance may last up to 48 hours [9, 13–15], although not all studies have been able to demonstrate this result [16–18]. This disparity in results may be due to differences among studies in the volume or intensity of exercise, the clinical or fitness status of the participants, or timing of the postexercise measurements.

Less than half of adults and children in the United States meet current physical activity recommendations [19, 20]. Although the long-term benefits of exercise are unquestioned, it is not yet clear how soon after starting an exercise program measurable impact on metabolic health can be detected in habitually sedentary people. The primary goal of the present study was to determine the magnitude of response in glucose tolerance and insulin sensitivity when young adults who are not habitual exercisers complete a single moderate-intensity exercise session appropriate for someone starting a structured physical activity program. The study was designed to measure the acute and residual effects of exercise performed either just prior to, or the day prior to a mixed meal challenge. Additionally, a preliminary experiment was performed to establish the reliability and validity of the mixed meal test as a tool for assessing glucose tolerance.

## 2. Methods

### 2.1. Study 1

To assess the reliability of the mixed meal test, a preliminary study was conducted with 18 people who completed two meal tests under similar conditions and an intravenous glucose tolerance test (IVGTT) for comparison. The group consisted of 14 females and 4 males who were 13–27 years old (3 children), 11 of whom were considered normal weight and 7 who were overweight based on body mass index (BMI) criteria. Four of the participants were recreationally active; the others were not regularly engaged in sports or exercise more than twice per week. The group was selected to be modestly diverse for age, body composition, and habitual physical activity so that the study outcomes would be generalizable. However, the participants could not have metabolic or other health conditions, or be using medications that would interfere with their safety or study outcomes.

Each participant completed an initial screening visit and three separate outpatient assessments of glucose tolerance/insulin sensitivity. The screening visit began with attainment of informed written consent from adults and consent and assent from each child and their parents in accordance with the university Institutional Review Board, which approved the study. Following a medical exam, body composition was measured using dual energy X-ray absorptiometry (DEXA, Lunar iDXA, GE-Healthcare, Fairfield, CT). The two meal tests and the IVGTT test were performed on three separate mornings at least one week apart at the University of Oklahoma General Clinical Research Center (GCRC). Before each trial, participants were instructed to maintain their normal activity pattern and to follow a consistent mixed diet for 3 days. Daily ambulatory activity during waking hours was recorded for 4 days before each trial with an accelerometer worn above the ankle (StepWatch 3, OrthoCare Innovations, Mountlake Terrace, WA). This monitor records the number of steps each minute and has high reliability and validity [21]. Data analysis included total step count and activity patterns based on step rates. A dietary log was used to record meals for 3 days.

On the morning of each trial, the participants reported to the GCRC at 07:00 AM following a 10-hour overnight fast. After the participant was quietly settled in a supine position, resting energy expenditure (REE) was measured for 30 minutes on both meal trials using an indirect calorimetry system with a flow-through canopy placed over the head (TrueOne 2400, ParvoMedics, Sandy, UT). An intravenous catheter was then placed in a forearm vein and kept patent with saline infusion for serial blood sampling. The mixed meal, consisting of a chocolate shake made from milk powder, milk cream, and chocolate syrup (2803 kJ, 45/40/15% of energy from carbohydrate/fat/protein, resp.), was consumed within 5 minutes. The start of the meal was designated as time 0 minutes. Blood collections were performed at −15 and −2 minutes (averaged and presented as the 0 minute fasting value), and again at 10, 20, 30, 40, 60, 90, 120, 150, and 180 minutes after the meal. The indirect calorimetry measurement was repeated for the last 20 minutes of each hour after the meal, with the final 15 minutes of each measurement used for analyses. The IVGTT trial was similar, starting with 30 minutes of supine rest, but without REE measurement. Intravenous catheters were placed in both arms for infusion and blood draw, respectively. After baseline blood collections at −15 and −2 minutes, glucose was infused at 0.3 g/kg body mass at 0 minutes and insulin at 0.12 pmol/kg body mass at 10 minutes [22], with blood sampling at 2, 3, 4, 5, 8, 10, 18, 19, 22, 28, 32, 40, 60, 70, 120, and 180 minutes.

### 2.2. Study 2

To assess the effect of a single exercise session on meal glucose tolerance, 11 young adults (7 women, 4 men) ages 20–30 years old were recruited from the local community. Participants were eligible if they had not been regularly engaged in organized sports or structured exercise programs for the previous 3 months and were not performing vigorous activity more than 30 minutes per session more than twice per week. Physical activity history was initially assessed by questionnaire and objectively measured prior to each trial with step monitors as in Study 1.

As in Study 1, the initial screening visit began with attainment of informed written consent, followed by a medical history and exam, and body composition measurement using DEXA. A submaximal treadmill walking test was performed to establish steady-state relationships among walking velocity, heart rate and oxygen uptake. Five-minute stages were performed at velocities ranging from 4.0–6.4 km/h at 0% grade. Similarly, submaximal and maximal responses were measured during an incremental workload test to volitional exhaustion on a stationary bicycle (Lode Corival, Groningen, The Netherlands). Three-minute stages were performed at 25, 50, and 75 watts, followed by increments of 15–20 watts/minute until fatigue. Finally, participants played the boxing game on the Nintendo Wii Sports (Nintendo of America, Redmond, WA) interactive video game system for about 10 minutes. During exercise, heart rate was continuously recorded with a chest-strap monitor (Polar Electro USA, Lake Success, NY) interfaced with an expired gas analysis system (Ultima Cardio2, MedGraphics, St. Paul, MN) as previously described [23].

Each participant returned to the GCRC between 07:00 and 07:30 AM following a 10-hour overnight fast for three morning trials conducted at least one week apart. On one trial, the participants performed no vigorous exercise for at least 3 days prior to the visit (No Ex trial). On a second trial, they completed a 45 minute bout of moderate intensity aerobic exercise in the afternoon, approximately 17 hours prior to the meal test (Prior Day Ex trial). On a third trial, the same exercise session was performed in the morning, after completing the baseline measures and approximately 30 minutes before the meal test (Same Day Ex trial). Thus, the tests were designed to measure the acute (Same Day Ex) and residual (Prior Day Ex) effects of a single exercise session in relation to the habitually low physical activity lifestyle pattern (No Ex) of these participants. Trial order was randomized. The exercise sessions were comprised of 15 minutes each of walking on the treadmill, stationary cycling, and video game boxing. This exercise protocol was selected to be appropriate and fun for novice exercisers, while involving multiple muscle groups. Walking and cycling workloads were adjusted to elicit 75% HRpeak. During boxing the participants were instructed to remain actively engaged in the game. On the Prior Day Ex trial, the exercise session was performed in the afternoon, with a time delay of 16.7 ± 0.1 hours between the end of the exercise and the start of the meal the following morning. On the Same Day Ex trial, the exercise was performed after the resting measurements were completed, with a time delay of 26 ± 2 minutes between the end of the exercise and the start of the meal. Blood collection and REE followed the same schedule as in Study 1.

All blood samples were separated into plasma or serum and stored at −70°C until analysis. Plasma glucose was measured by the glucose oxidase method (2300STAT Plus, Yellow Springs Instruments, Yellow Springs, OH). Serum insulin and C-peptide were measured using Elisa kits from Millipore (St. Louis, MO). Nonesterified fatty acids (NEFAs) were measured in serum with an enzymatic colorimetric assay (Wako Chemicals, Richmond, VA). The postmeal peak and area under the curve (AUC) for each of these outcomes was used for analyses. The glucose and insulin values from the meal tests were also used to calculate the whole body insulin sensitivity index (ISI) described by Matsuda [24]. Insulin sensitivity (SI) from the IVGTT was calculated using the computer minimal modeling approach [22, 25] and the MINMOD Millennium software package from the Berman Laboratory. Quantification of serum triglycerides and total-, HDL-, and LDL-cholesterol; and C-reactive protein was performed by the Clinical Chemistry Laboratory of the Oklahoma Veterans Administration Hospital (Oklahoma City) using validated enzymatic assays (Synchron Systems, Beckman Coulter, Brea, CA).

### 2.3. Statistical Analyses

For Study 1 the within-subject coefficient of variation (CV) and intraclass correlation for the primary outcomes were calculated according to Hopkins [26] as measures of the reproducibility of responses between the two meal trials. For Study 2, statistical analyses were performed using repeated measures analysis of variance with Bonferroni post-hoc tests to locate pairwise differences among trials as appropriate. Strength of association among selected variables was calculated using Pearson's correlation. For all tests, significance was accepted at *P* < 0.05. Summary data are presented as mean ± SEM except for participant characteristics as noted.

## 3. Results

Participant characteristics for both studies are presented in Table 1. None of the participants had metabolic syndrome, hypertension, or hyperlipidemia.

### 3.1. Study 1

Due to missing data, step activity results were available for only 15 of the participants. The monitors were worn for 14.5 ± 0.6 hours/day for 3.4 ± 0.2 days, recording an average of 10,280 ± 569 steps/day. The amount of time with no or low (<30 steps/minute) activity was 86 ± 1%. There was no difference in activity volume or pattern across trials.

**Figure 1:**
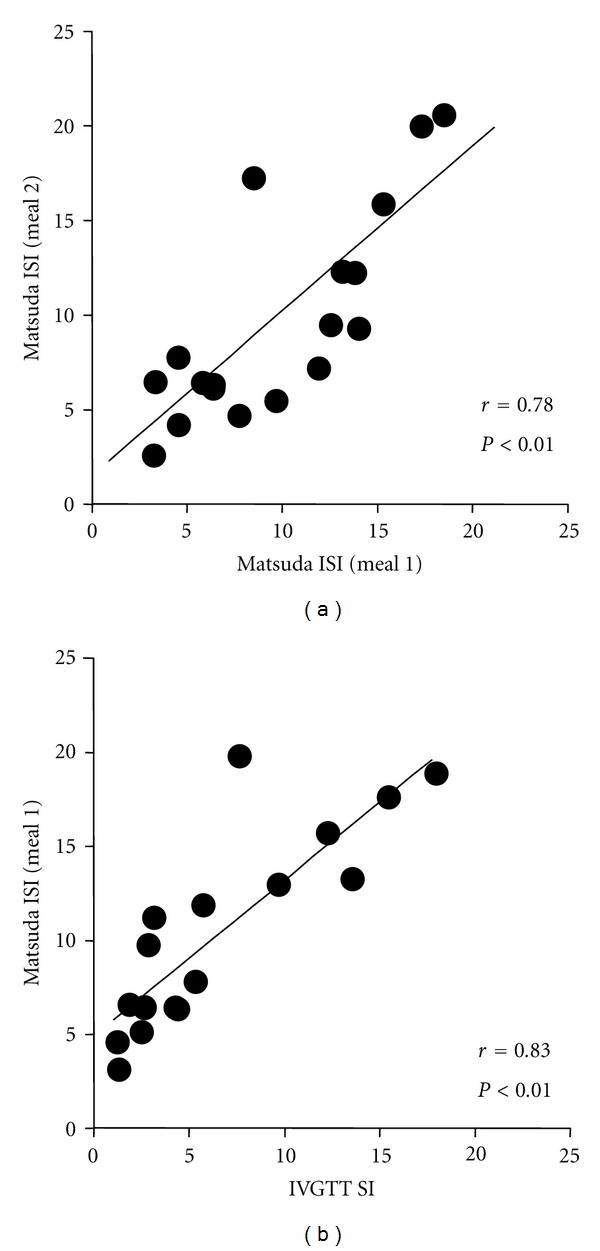
Insulin sensitivity in young healthy people in Study 1. (a) Correlation between the Matsuda whole body insulin sensitivity index (ISI, arbitrary units) measured during two identical mixed meal tests. (b) Correlation between the minimal model-derived estimate of insulin sensitivity (SI, units = 10^−4^/min ×  *μ*U/mL) during an intravenous glucose tolerance test (IVGTT) and the ISI from the first mixed meal test.

On the two meal trials, there were no differences in the fasting, peak, or time to peak values for glucose, insulin, C-peptide or fatty acids (not shown). The test-retest correlation between meal tests and the CV for the AUCs, respectively, were for glucose: *r* = 0.87, CV = 4%, for insulin: *r* = 0.83, CV = 15%, for C-peptide: *r* = 0.74, CV = 15%, and for fatty acids: *r* = 0.44, CV = 26%. The average Matsuda ISI values were 9.27 ± 1.17 and 9.33 ± 1.32 on the two meal tests, with a CV of 20%. The average value for SI from the IVGTT was 5.92 ± 1.25 10^−4^/min ×  *μ*U/mL. The test-retest correlation for the meal ISI is shown in Figure 1, along with the correlation between the meal ISI (test 1) and the SI measured during the IVGTT. Although both of the correlation coefficients in Figure 1 fall within the “large effect” range according to Cohen [27], an *r*-value above 0.90 is a commonly accepted goal for test-retest reliability [26]. Notably, if the values for the participant with the highest variance between tests was removed, the test-retest correlations improved to *r* = 0.91 for glucose AUC, *r* = 0.89 for insulin AUC, and *r* = 0.86 for meal ISI, and the correlation between meal ISI and IVGTT SI improved to *r* = 0.91, respectively. Despite the negative influence on the results by the outlier participant, no data were removed from the final analyses since there were no obvious explanations, such as technical error or noncompliance with the protocol, for the variable results.

The meal test results were used to calculate the statistical power and sample sizes for future studies. With an expected test-retest correlation coefficient of *r* ≥ 0.80 and the CVs listed above, the sample size used in Study 2 of *N* = 11 afforded 80% power with the alpha error level at 5% to detect significant differences between trials of 5% for glucose AUC, 20% for insulin AUC, and 17% for c-peptide AUC.

There was no difference in basal REE between meal studies (4.51 ± 0.23 versus 4.48 ± 0.22 kJ/min, resp., test-retest *r* = 0.90, CV = 6%). EE was elevated (*P* < 0.01) throughout the postprandial period by 25 ± 3, 18 ± 2 and 13 ± 2% at 60, 120, and 180 minutes, respectively, with no difference between trials. The total EE over the 3-hour measurement time was 887 ± 34 kJ in Meal 1 and 901 ± 38 kJ in Meal 2, respectively (test-retest *r* = 0.96, CV = 3%). Carbohydrate oxidation accounted for 51 ± 3% of EE at baseline and was increased during the postprandial period (87 ± 5, 67 ± 4, 57 ± 3% at 60, 120, and 180 minutes, resp.), with no difference between trials.

### 3.2. Study 2

All participants wore the step monitors, although after accounting for missing days the average recording time was 3.5 ± 0.2 days before each test, recording 7635 ± 559 steps over 13.0 ± 0.4 hours per day with no difference across trials. Time spent in no activity or low-intensity activity accounted for 89 ± 1% of daily monitoring time. During the Prior Day Ex and Same Day Ex trials, the average exercise HR was 76 ± 4% and 75 ± 4% of peak, respectively, with no difference between trials or across exercise modes (72, 77, and 78% HRpeak for treadmill, cycling, and boxing resp.). The estimated total energy expenditure during exercise was 1183 ± 41 kJ on both exercise trials.

**Figure 2:**
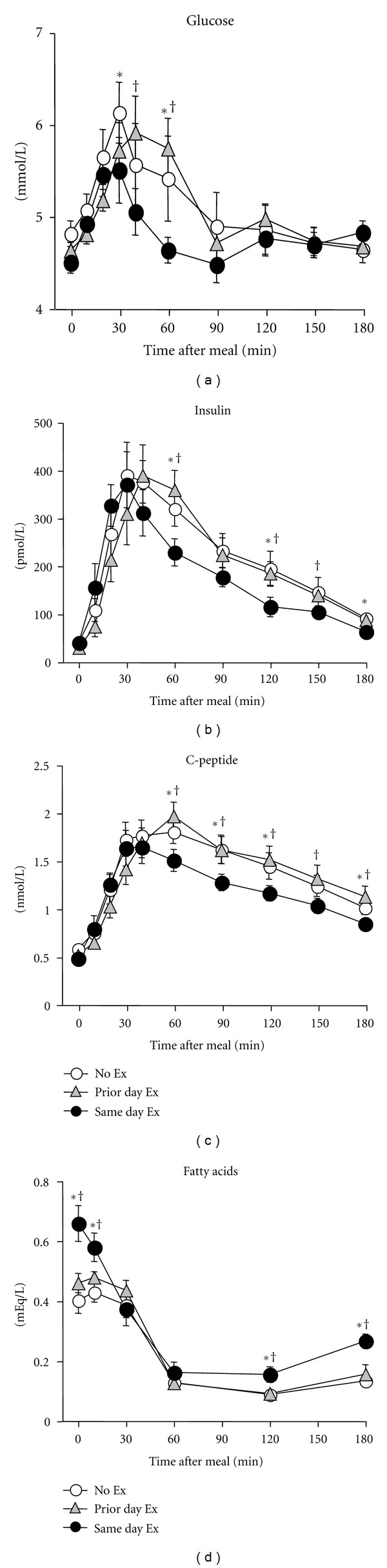
Postmeal responses in glucose, insulin, C-peptide, and nonesterified fatty acids in Study 2. Values shown as mean ± SEM for 11 people. *No Ex different from Same Day Ex trial; ^†^Prior Day Ex different from Same Day Ex, *P* < 0.05.

**Figure 3:**
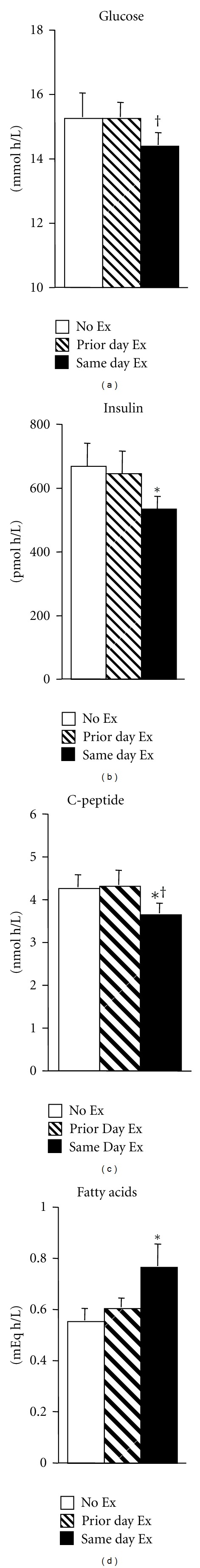
Area under the curve for glucose, insulin, C-peptide, and fatty acids during the post meal period. Values shown as mean ± SEM for 11 people. *No Ex different from Same Day Ex trial; ^†^Prior Day Ex different from Same Day Ex, *P* < 0.05. There were non significant trends for differences between Prior Day Ex versus Same Day Ex insulin (*P* = 0.059) and fatty acids (*P* = 0.092).

As shown in Figure 2 glucose, insulin, and C-peptide remained elevated above baseline and fatty acids were suppressed throughout most of the 3-hour postmeal measurement period. There were no differences between the No Ex and Prior Day Ex trials for these outcomes. However, from 30 minutes and onward after the start of the meal glucose, insulin and C-peptide were lower or showed a trend to be lower, on the Same Day Ex trial versus the No Ex and/or Prior Day Ex trial. For fatty acids, higher values were present in the Same Day Ex trial versus the other two trials both at the start of the meal and the end of the postprandial observation period. As shown in Figure 3, the Same Day Ex trial resulted in reductions in the AUC for glucose (6%), insulin (20%), and C-peptide (14%), and an increase for fatty acids (38%) relative to the other two trials, which were not different from one another. However, those differences among trials were not fully reflected by the Matsuda ISI value; although the ISI was 18% higher on the Same Day Ex trial versus No Ex (9.35 ± 1.56 versus 7.90 ± 1.25), this difference was not statistically significant. ISI on the Prior Day Ex trial was 9.09 ± 1.08 and also not different from the other two trials.

**Figure 4:**
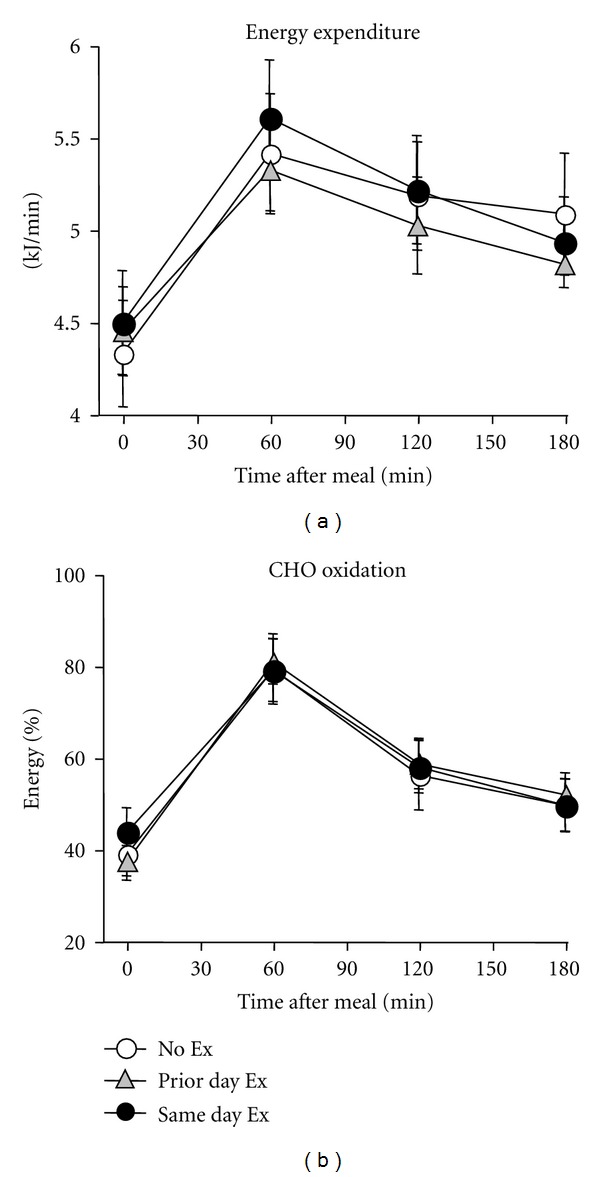
Postmeal responses in energy expenditure and fuel oxidation. Energy expenditure and the relative carbohydrate (CHO) oxidation were increased throughout the postmeal period relative to baseline but did not differ among trials.

The average baseline REE (Figure 4) on the No Ex trial was 4.20 ± 0.09 kJ/min, which increased 26% at 1 hour after the meal and remained 20% and 18% elevated at 2 and 3 hours after the meal (*P* < 0.01 for postmeal comparisons with baseline value in each trial). Carbohydrate oxidation rose from 38% at baseline to 78% at 1-hour postmeal on the No Ex day, remaining above the fasting baseline through 3 hours (Figure 4). The total 3-hour carbohydrate oxidation was 31 ± 1 grams. Fasting and postprandial EE and fuel oxidation did not differ among trials.

## 4. Discussion

The goals of this investigation were to establish the reliability of a mixed meal test for assessing glucose tolerance and insulin action, and to measure the acute and residual impact of a single session of endurance exercise on meal glucose tolerance in habitually sedentary, but healthy young adults. Results of the first study demonstrated acceptably high test-retest reliability for postprandial glycemic and insulinemic responses and strong correlation between the meal ISI and IVGTT SI, while the main finding of the second study was that meal glucose control was improved following a moderate intensity exercise session compared to a trial without prior exercise. However, the beneficial effect of exercise was evident only when completed within an hour before the mixed meal test but not when the participants exercised the day prior (~17 hours) to the meal. Thus, for habitually sedentary young adults, insulin sensitivity was acutely responsive to a volume and intensity of exercise that is consistent with current recommendations for adults. The finding that the improvement was transient, however, highlights the importance of engaging in frequent exercise to promote metabolic health.

The results of Study 1 demonstrated that the postprandial glucose and insulin responses to the mixed meal test had acceptably high reproducibility between tests, and validity when compared to the IVGTT. Thus, the mixed meal can be used to assess the impact of exercise with a less-intensive method than the IVGTT or euglycemic hyperinsulinemic clamp but is perhaps more physiological than the oral glucose tolerance test [28]. We recognize, however, that daily glycemic regulation is also dependent on the size and digestibility of the meal, the influence of incretin hormones, and other variables that were not explored in the current investigation. To our knowledge, there are no other similar reports that provide the within-subject variability results in healthy young people for a standard liquid mixed meal test, so the data acquired were useful for predicting the statistical power in Study 2 or other future investigations. Though there are many studies that have compared various parameters of insulin sensitivity and glycemic control among the different types of tests (e.g., fasting tests, oral glucose, IVGTT, insulin/glucose clamps), there are many fewer studies that report the reproducibility of these tests [28]. Nevertheless, results in the current study compare favorably with prior reports. For example, the CV for the Matsuda ISI from two oral glucose tolerance tests was 14–20% in adults across a range of glucose tolerance [29]. For the IVGTT, the CV for calculated SI was 20–27% for young men in one study [30], while another study reported that the CVs for insulin and C-peptide AUCs were 22% and 19%, respectively (variation for glucose AUC and calculated SI was not given) [31]. The reproducibility of the euglycemic hyperinsulinemic clamp appears to be higher than oral tests or the IVGTT, as the CV for repeat measures of glucose disposal rate in adults was 9–12% [32]. Thus, our finding that CVs for glucose, insulin, and c-peptide AUC and Matsuda ISI were 4–20% across two meal tests is not much different than prior reports. We determined that physiologically important changes in those outcomes in response to interventions like exercise should be reliably detectible with manageable samples sizes of 10–20 people depending on the magnitude of the target effect size. Additionally, the correlation between the meal ISI and the IVGTT SI in the current study (Figure 1) is similar to or greater than reported correlations between estimates of insulin sensitivity attained from euglycemic hyperinsulinemic clamp versus IVGTT [33] or oral glucose tolerance test [24]. Notably, we did not provide a fixed diet or admit the participants as inpatients to the clinical research center prior to their studies, which might have allowed for even lower variation in results by tighter control of diet and physical activity. However, we confirmed that physical activity levels were similar prior to each test with step monitors and gave repeat instructions to follow the same diet prior to each trial.

In Study 2, the exercise energy expenditure of ~1180 kJ during the 45-minute session was lower than typically used (~1250–3350 kJ) in prior single exercise session studies performed with adults [9, 13, 15–18, 34]. The lower exercise volume may explain the relatively modest improvement in glucose, insulin, and C-peptide AUC on the Same Day Ex trial and failure for the exercise to exert even a partial effect on the Prior Day Ex trial, or carbohydrate oxidation on either exercise trial. Nevertheless, the results are generalizable to the population of apparently healthy but untrained young adults since the exercise session was designed to be feasible and enjoyable for people who were unaccustomed to regular structured physical activity, using three exercise modes that incorporated both upper and lower body movements. This type of session would be appropriate for people starting an exercise program, with the goal of progressively increasing the duration and intensity toward the current recommendation of 150 minutes per week of moderate-to-vigorous activity [35].

In animals and humans insulin and noninsulin mediated pathways for glucose uptake in skeletal muscle are activated for several hours after exercise, although the specific pathways and how they are regulated have not been fully elucidated [12, 36]. In some [9, 13–15], though not all [16–18] previous human studies the effect of a single exercise session on insulin sensitivity was reported to last 12–48 hours. Notably, in all of those reports the postexercise measurement was performed at only one time point, so the time course of insulin sensitivity response has not been well described. In one prior study, untrained adults performed 45 minutes of stair-climbing exercise (energy expenditure estimated to be ~1300 kJ) and it was reported that insulin-mediated glucose disposal was increased by ~25% two days later [9]. To our knowledge that study was the only one to report such a persistent improvement in insulin sensitivity in a group of untrained people performing a moderate volume of activity. Previously, Mikines et al. [13] showed that insulin action was increased for 48 hours after a single exercise session, but the participants were described as recreationally active and were able to complete 60 minutes of cycling at 150 W (~2600 kJ), a workload that exceeds the average peak power output attained by the untrained people in the current investigation. Likewise, insulin sensitivity was increased 12 hours after exercise compared to a no-exercise trial in young men who performed multiple sets of leg resistance exercise [14], and in young overweight men and women who performed an 84-minute cycling session (~3000 kJ) [15]. In agreement with the current study though, insulin sensitivity was not significantly improved in three other investigations in which adults completed 60–75 minutes of moderate intensity cycling, walking and/or rowing (~1250–2100 kJ) 12–17 hours before the postexercise testing was performed [16–18]. Thus, exercise volume and/or intensity are likely to be important, but not the only regulators of the magnitude and duration of insulin action response.

Additional factors responsible for variation among studies that measured postexercise insulin sensitivity may be the type and amount of nutrients consumed between the end of exercise and the measurement of insulin action, and the characteristics of the group under study, such as their age, anthropometric differences, medical or physical fitness status, and family history of diabetes. For example, recent work in humans and prior studies in rodents showed that the magnitude and/or duration of the postexercise stimulation of insulin sensitivity can be enhanced by consuming a low-carbohydrate diet and/or maintaining a short-term energy deficit [12, 15, 34, 37]. The selection of diet and exercise conditions to enhance metabolic health is becoming increasingly important in light of the growing prevalence of obesity and sedentary lifestyle. In study 2 of the present investigation, all of the participants were normal weight according to BMI standards, and metabolic flexibility was evident from the shift in fuel oxidation from predominantly fat following overnight fast to high utilization of carbohydrate after the meal was consumed. It is not yet clear if a different response following a single exercise session could be expected in people with obesity, diabetes, or related metabolic conditions. Previous studies showed that overweight people either did [15] or did not [17] demonstrate improvement in insulin action 12–17 hours after exercise. The study by Perseghin et al. [9] showed that insulin-mediated glucose uptake was similarly improved after a single exercise session in people with or without insulin resistance. In contrast, we [38] and others [39, 40] have shown that adults who have parents or siblings with type 2 diabetes may have an impaired ability to improve insulin sensitivity following either short-term (3–9 days) or longer-term (26 weeks) aerobic exercise training. The mechanism for this blunted response is not yet resolved; there is evidence, for example, for specific gene polymorphisms [39, 40] and mixed evidence to support a role for mitochondrial oxidative capacity [38–40]. Since there are relatively few studies directly comparing how a single exercise session, or only a few training sessions at the start of an exercise program, affects insulin sensitivity in people of different ages, body fatness, physical fitness, or metabolic health status, the impact of these variables is not yet resolved. Another question that has not yet been adequately addressed is how long the beneficial effect of a single exercise session lasts in previously inactive, untrained people. As noted, we chose the exercise session in the current investigation because it was feasible for people unaccustomed to regular daily exercise, but there may be different time course of response depending on the duration, intensity, or mode of physical activity performed.

In summary, the results of the present study demonstrate that young adults with low aerobic fitness and habitually low physical activity respond to a single moderate intensity bout of exercise with an acute improvement in insulin sensitivity when measured within 3 hours of exercise with a mixed meal test, but that this effect is no longer evident when measured 17 hours after exercise. Whether and how the magnitude of this response can be enhanced by modification of the exercise conditions, diet, or other manipulations is not yet known but could have important effects on the health of sedentary people. These results highlight the need to encourage young adults to engage in daily moderate-to-vigorous physical activity and the immediate beneficial impact of exercise on metabolic function.

## Figures and Tables

**Table 1:** Participant characteristics.

	Study 1	Study 2
Age, y	24 ± 4	26 ± 3
Body mass, kg	72.9 ± 3.9	65.8 ± 9.0
Height, m	1.70 ± 0.09	1.70 ± 0.10
BMI, kg/m^2^	25.0 ± 1.1	22.8 ± 0.9
Body fat, kg	24.2 ± 11.4	19.3 ± 3.7
Body fat, %	32.4 ± 2.0	29.8 ± 6.5
Lean mass, kg	45.3 ± 8.1	43.6 ± 8.7
Peak bike power, watts	n/a	145 ± 43
Peak VO_2_, mL/kg/min	n/a	26.4 ± 5.9
Peak heart rate, beats/min	n/a	179 ± 15
Total cholesterol, mmol/L	3.98 ± 0.99	4.03 ± 0.90
HDL cholesterol, mmol/L	1.21 ± 0.37	1.11 ± 0.33
Triglycerides, mmol/L	0.74 ± 0.39	0.89 ± 0.31
C-reactive protein, nmol/L	18.4 ± 19.9	11.0 ± 5.2
Glucose, mmol/L	4.7 ± 0.2	4.8 ± 0.5
Insulin, pmol/L	41 ± 28	41 ± 27
Systolic blood pressure, mmHg	109 ± 9	112 ± 10
Diastolic blood pressure, mmHg	68 ± 8	62 ± 7

Values are mean ± SD for 14 females and 4 males in Study 1 and 7 females and 5 males in Study 2. Body composition determined by DEXA. Peak exercise responses were measured in Study 2 during a bicycle test to volitional exhaustion (not performed in Study 1). Blood test results are from a fasting sample collected during the first meal test in Study 1 and the No Ex trial in Study 2.
